# RhoC: a fascinating journey from a cytoskeletal organizer to a Cancer stem cell therapeutic target

**DOI:** 10.1186/s13046-019-1327-4

**Published:** 2019-07-24

**Authors:** Pavana Thomas, Annapurna Pranatharthi, Cecil Ross, Sweta Srivastava

**Affiliations:** 1 Translational and Molecular Biology Laboratory (TMBL), St. John’s Research Institute (SJRI), Bangalore, 560034 India; 2 School of Integrative Health Sciences, The University of Trans-Disciplinary Health Sciences and Technology (TDU), Bangalore, 560064 India; 3 Rajiv Gandhi University of Health Sciences (RGUHS), Bangalore, 560041 India; 4 National Centre for Biological Sciences (NCBS), Bangalore, 560065 India; 5 Translational and Molecular Biology Laboratory (TMBL), Department of Medicine, St. John’s Medical College Hospital (SJMCH), Bangalore, 560034 India; 6 Translational and Molecular Biology Laboratory (TMBL), Department of Transfusion Medicine and Immunohematology, St. John’s Medical College Hospital (SJMCH), Bangalore, 560034 India

**Keywords:** RhoC, Cancer stem cells, Tumor phenotypes, Therapy resistance

## Abstract

Tumor heterogeneity results in differential response to therapy due to the existence of plastic tumor cells, called cancer stem cells (CSCs), which exhibit the property of resistance to therapy, invasion and metastasis. These cells have a distinct, signaling network active at every stage of progression. It is difficult to envisage that the CSCs will have a unique set of signaling pathways regulating every stage of disease progression. Rather, it would be easier to believe that a single pivotal pathway having significant contribution at every stage, which can further turn on a battery of signaling mechanisms specific to that stage, would be instrumental in regulating the signaling network, enabling easy transition from one state to another. In this context, we discuss the role of RhoC which has contributed to several phenotypes during tumor progression.

RhoC (Ras homolog gene family member C) has been widely reported to regulate actin organization. It has been shown to impact the motility of cancer cells, resultantly affecting invasion and metastasis, and has contributed to carcinoma progression of the breast, pancreas, lung, ovaries and cervix, among several others. The most interesting finding has been its indispensable role in metastasis. Also, it has the ability to modulate various other phenotypes like angiogenesis, motility, invasion, metastasis, and anoikis resistance. These observations suggest that RhoC imparts the plasticity required by tumor cells to exhibit such diverse functions based on microenvironmental cues. This was further confirmed by recent reports which show that it regulates cancer stem cells in breast, ovary and head and neck cancers. Studies also suggest that the inhibition of RhoC results in abolition of advanced tumor phenotypes.

Our review throws light on how RhoC, which is capable of modulating various phenotypes may be the apt core signaling candidate regulating disease progression. Additionally, mice studies show that RhoC is not essential for embryogenesis, giving scope for its development as a possible therapeutic target. This review thus stresses on the need to understand the protein and its functioning in greater detail to enable its development as a stem cell marker and a possible therapeutic target**.**

## Background

**Fig. 1:**
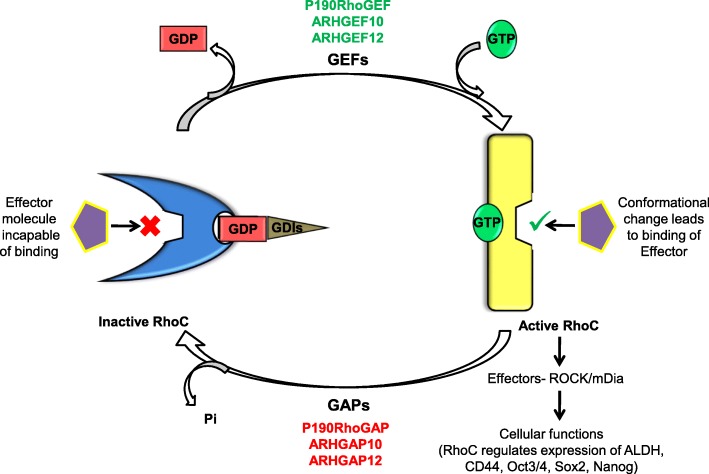
Cycling of Ras homolog gene family member C (RhoC) between active and inactive forms: The switching of RhoC between the inactive GDP-bound form to the active GTP-bound form is regulated by Guanine Nucleotide Exchange Factors (GEFs), GTPase Activating Proteins (GAPs) and Guanine Dissociation Inhibitors (GDIs). Binding to GTP changes the conformation of the molecule, thus allowing the binding of various downstream effectors of RhoC like Diaphanous Related Formin (mDia) and Rho Associated Coiled-Coil Containing Protein Kinase (ROCK), thereby facilitating various downstream signaling pathways

Despite major advances in molecular and diagnostic sciences, and the emergence of personalized treatment, challenges remain due to the non-availability of personalized medicine across all cancers and the ever-evolving nature of this form of therapy. Therefore, the study and exploration of signaling pathways has intensified in the quest for novel therapeutic targets. The role of Notch, Wnt, Tumor Growth Factor-beta (TGF-beta) and Nuclear Factor kappa-light-chain-enhancer of activated B cells (NFκB) amongst several other signaling pathways, has been well studied over the years and across several tumors. Consequently, several candidates (like Epidermal Growth Factor Receptor (EGFR) for lung cancer) have been developed as molecular targets for personalized medicine. Another signaling pathway that has been shown to contribute extensively to tumor progression in several tumor types is the Ras homolog gene family member C (RhoC) signaling pathway. RhoC belongs to the Rho family of small Guanosine Triphosphatases (GTPases) [1]. Rho GTPases are small signaling G-proteins that regulate cytoskeletal organization and thus affect multiple cellular functions, including cell motility, polarity and division by switching between the Guanosine Triphosphate (GTP) and Guanosine Diphosphate (GDP) bound states, as shown in Fig. 1 [2–5]. This switch in states is tightly regulated by RhoGAPs (Rho GTPase Activating Proteins), RhoGEFs (Rho Guanine Exchange Factors) and RhoGDIs (Rho Guanine Dissociation Inhibitors) [6]. RhoGAPs support the intrinsic GTPase activity of RhoGTPases, converting them from the GTP-bound state to GDP-bound, thereby leading to their deactivation [7]. RhoGEFs on the other hand help maintaining RhoGTPases in the active state by facilitating their switch from the GDP-bound form to the GTP-bound form [8]. The third regulator protein, the RhoGDIs, stabilize the RhoGTPases in the GDP form, consequently playing an important role in determining localization of the protein [9]. Active forms of the protein, GTP-bound, regulate the actin cytoskeleton, cell cycle, membrane trafficking and transcription [10]. Significantly, the activity of each RhoGTPase is governed by regulators specific to each of them, with reports suggesting that the activity of RhoC in particular is regulated by GEFs like p190RhoGEF, ARHGEF10, ARHGEF12 and GAPs like p190RhoGAP, DLC1 to name a few [11–14]. Although the Rho isoforms have more than 90% sequence homology with each other and are known to regulate actin organization, several studies have proven that they have vastly distinct functions [15]. For example, RhoA and RhoC localize in the cytoplasm while RhoB localizes to the endosomal membrane [16]. In mouse embryonic fibroblasts, RhoA is dispensable for actomyosin regulation; however, it is important for mitosis [17, 18]. RhoC, too, has been shown to be responsible for cytoskeletal reorganization and cellular motility. Nevertheless, RhoA and RhoC have distinct roles in invasion, as they act through different targets [19]. In the context of viral infections, the process of cell contraction through the viral protein F11, is seen to be dependent on ROCK signaling via activation by RhoC and not RhoA. Additionally, this effect is seen to be abrogated by recruitment of Pak6 to the cellular membrane by another RhoGTPase, RhoD [20]. An interesting study by Hakem et al. showed that RhoC is dispensable for embryogenesis but is essential for metastasis [21]. While RhoC has an important contribution to metastasis, RhoB has been reported, using mice models, to be a tumor suppressor [22]. A study in colorectal cancer indicated a strong correlation of both RhoA and RhoC in metastasis and invasion [23], whereas other studies in breast and colon cancer have suggested that RhoA often inhibits cell invasion, while RhoC, on the other hand, enhances cell invasion [24, 25]. In 1989, Chardin and colleagues showed that RhoC affects actin microfilaments in Vero cells [26]. Following this report, there have been incessant efforts to understand the role of this molecule in both physiological and patho-physiological conditions. Here, we provide a comprehensive account of the work done on this molecule in the context of cancer progression and resistance to therapy, followed by an outline of the work that remains to be performed to enable better understanding of the working of this protein within the cell.

### RhoC in tumor phenotypes and molecular pathways

The role of RhoC in carcinoma progression has been extensively clarified by several research groups over the years. The first report, which suggested that RhoC contributed to progression of cancer, was by Suwa et al. in the year 1998. This group investigated changes in the expression levels of the Rho family of genes—RhoA, B and C in pancreatic ductal carcinoma. It was discovered that the expression of the RhoC gene was significantly higher in metastatic tumors than in primary tumors, whereas RhoA and RhoB did not show significant changes in expression under these conditions. Also, increased RhoC expression significantly correlated with poor prognosis of patients, unlike RhoA and RhoB, which showed no such correlation [27]. Following this study, several other groups reported the role of RhoC in numerous other cancers, including those of the breasts, skin, ovaries, liver and head and neck, among several others [28–33]. The increased expression of RhoC is therefore positively correlated to poor prognosis. However, activation of the molecule is necessary to enable its downstream effects. MyoGEF, a molecule responsible for activation of RhoA and RhoC was found to regulate both polarity and invasive phenotypes of MDA-MB-231 (an invasive breast cancer cell line) [34]. On the contrary, p190RhoGAP which converts GTP-bound Rho to the inactive GDP-bound form, is associated with reduced proliferation, migration and invasion in breast and pancreatic cancer models, thus acting as the antithesis of MyoGEF in this context [35, 36]. The role of RhoC as a transforming oncogene was postulated by van Golen et al. This group demonstrated that the stable transfectants of human mammary epithelial cells over-expressing RhoC not only gained tumorigenic properties but were also highly invasive [32]. In the year 2013, Xie et al. showed that stable transfection of the RhoC expression vector into a normal hepatocyte cell line, imparted tumor phenotypes like proliferation, anchorage-independent growth, migration, invasion, increased expression of matrix metalloproteases like MMP2 and MMP9, and elevated levels of Vascular Endothelial Growth Factor (VEGF), further cementing RhoC’s role as an oncogene [37]. Additionally, RhoC was found to have a positive association with dedifferentiation and the phosphorylated form of p70s6k, a protein well known for its role in promoting survival and proliferation, thus making it a probable marker for carcinogenesis and the progression of ovarian epithelial carcinoma [38].

MicroRNAs (miRNAs), which have diverse cellular functions, have been shown to regulate RhoC expression. Chen X et al., showed in 2015 that increased miR-93-5P (specific to RhoC) resulted in decreased tumorigenesis and the progression of epithelial ovarian carcinoma [39]. Another microRNA, miR-10b, inhibits the translation of homeobox D10. This process leads to increased RhoC expression, resulting in increased invasion and metastasis of breast cancer [40]. The tumorigenesis and progression of ovarian epithelial carcinoma was also seen to be inhibited by miR 106b, which binds to the 3′ UTR of RhoC [41]. Long non-coding RNA (lncRNA) TDRG1 increases RhoC expression, consequently leading to tumorigenesis in the ovarian epithelial carcinoma model via miR-93 [42]. Similarly, over-expression of lncRNA ABHD11-AS1 correlates with the progression of epithelial ovarian carcinoma by regulating RhoC [43]. Signaling pathways regulated by RhoC are also involved in regulating the expression of certain lncRNAs. The expression of HOTAIR, an lncRNA known to be a negative prognostic marker, is under the influence of RhoC-ROCK signaling in breast cancer cells [44]. On the other hand, the proliferation, invasion and metastasis of gastric cancer was blocked by miR-493, which was proven to directly target RhoC [45]. Likewise, miR-372 over-expression led to G1 arrest and apoptosis, along with a suppression of tumor growth and the metastasis of endometrial carcinoma via inhibition of RhoC [46].

The switch from a locally confined tumor to an invasive, metastatic form is the most damaging alteration in a tumor; allowing it to disseminate, eventually leading to a poor prognosis. Epithelial to mesenchymal transition (EMT) is a prerequisite to metastasis [47–51]. Interestingly, DNA array analysis of metastatic melanoma cells revealed that RhoC was important for metastasis [52]. RhoGTPases are also known to regulate the activity of myocardin-related transcription factors MRTFA/B, which are upstream of genes necessary for metastasis [53]. Inhibition of MRTF using a pharmacological inhibitor CCG-203971, led to decreased lung metastases in mice injected with the highly invasive, RhoC overexpressing melanoma cell line SK-Mel-147 [54].Bellovin et al. showed that Ets-1 increases RhoC expression in LIM1863 colon cancer cells, resulting in increased EMT and cell migration [24]. Interestingly, Zhou X et al., demonstrated that HIF (Hypoxia Inducible Factor), a protein known to be associated with abnormal growth and invasion, acts via trasncriptionally altering the RhoC-ROCK1 pathway in the pancreatic cancer model [55]. RhoC also regulates EMT in cervical cancer, wherein the inhibition of Notch1 and RhoC resulted in the abolition of actin stress fiber formation and fibronectin expression, the two important changes associated with EMT [56]. The Rho proteins regulate cytoskeletal organization, and true to its nature, RhoC has been shown to regulate actin organization in tumors resulting in enhanced migration, invasion and metastasis [21, 24, 57–60]. Significantly, using stable benign breast epithelial cell lines with inducible RhoA and RhoC expression, Sarah Lang et al. have shown, that RhoC and not RhoA, is indispensable for invasion [61].

The close association between TGF-β1 and RhoC has been reported in several tumors. RhoC, known to play an important role in rearrangement of the cytoskeleton, has been implicated in EMT, invasion and metastasis of lung adenocarcinoma cells when induced by TGF-β1. Moreover, the down-regulation of RhoC using shRNA abolished TGF-β1-mediated EMT induction [62]. Similarly, in ovarian epithelial carcinoma cells, RhoC has been shown to mediate EMT that is stimulated by TGF-β1 and VEGF [63]. A similar study performed on the cervical carcinoma model demonstrated that RhoC is necessary for TGF-β1 driven EMT [64]. Notably, it has been postulated that tumor cells disseminate either as single cells or move collectively. Using intravital imaging, Giampieri and group showed that TGF-β switches breast cancer cells from cohesive to single cell motility, which is essential for intravasation, by transcriptionally reprograming tumor cells, thereby leading to alteration in the expression of several genes, including RhoC [65].

In cervical cancer, Notch1 has been proven to regulate RhoC leading to changes in migration and invasion [56]. Similarly, stromal cell-derived factor-1 (SDF-1) was seen to modulate Jurkat cell migration through the RhoC-ROS pathway [66]. Using the SUM-149 Inflammatory Breast Cancer (IBC) cell line, Joglekar et al., have reported that caveolin-1 regulates RhoC-mediated invasion by activation of Akt-1 [67]. In the colon cancer model, HOXD10 and RhoC were shown to negatively correlate with each other in both patient specimens and cell lines. Further analysis revealed that increased HOXD10 led to suppression of the MAPK and AKT pathways known to regulate RhoC [68]. The interaction of FMNL-3 with RhoC was seen to lead to increased MMP2, MMP9 and VEGF, consequently leading to increased invasion in colon cancer cell lines [69]. The knockdown of RhoC in cholangiocellular carcinoma cells on the other hand, resulted in the suppression of invasion and migration [70]. On similar lines, YMO1, a protein belonging to the Yurt and mosaic family, was seen to reduce the invasion and metastatic ability of hepatocellular carcinoma cells, by targeting RhoC [71].

There are several pathways, which are regulated by RhoC, that contribute to carcinoma progression and maintenance. RhoC alters the Mitogen Activated Protein Kinase (MAPK) and Phosphoinositide 3 kinase**/**AKT Serine Threonine Kinase (PI3K/AKT) pathways to regulate invasion [72, 73]. Interestingly, while RhoC is an important player in the motility of inflammatory breast cancer (IBC) and melanoma, it does not contribute to motility in prostate cancer cell lines, such as PC-3. However, RhoC does regulate the invasion of PC-3 [74]. RhoC has also been shown to activate the Protein-Tyrosine Kinase 2 (PYK2) pathway in prostate cancer, consequently leading to metastasis in prostate cancer [75]. Immunohistochemical analysis of RhoC expression in this study showed a significant correlation between both lymph node and distant metastases and the activation of Matrix Metalloprotease 2 (MMP2) and Matrix Metalloprotease 9 (MMP9). Further, antibody array analysis showed that RhoC activated several kinases, including MAPK, Focal Adhesion Kinase (FAK), AKT and PYK2. RhoC also regulates Formin-like 3 (FMNL3) mediated cell migration and invasion as it is involved in polarized migration [19]. In another study, RhoC has been shown to stimulate alpha5 integrin expression and the Src-dependent activation of p130 Crk-associated substrate/Ras-Related C3 Botulinum Toxin Substrate 1 (Cas/Rac1) signaling [76]. RhoC also controls cofilin activity to modulate actin organization, resultantly affecting invasion and invadopodia formation [12, 77]. Table 1 summarizes the pathways in which RhoC has been implicated. These and several more studies elucidate the mechanisms of RhoC-mediated regulation of cancer phenotypes.

**Table 1:** The various signaling pathways in cancer via which RhoC operates

Signaling Pathways in Cancer	Reference
Notch1	Srivastava S et al., 2010 [40]
TGF-β1	He X et al., 2015 [48]
PI3K-Akt	Ruth MC et al., 2006 [53]
Pyk2	Iiizumi M et al., 2008 [56]
Cas/Rac1	Arpaia E et al., 2011 [57]
VEGF	Hoeppner LH et al., 2015 [60]
MMP9	Zhao Y et al., 2010 [61]
EGFR	Tumur et al., 2015 [62]

**Fig. 2:**
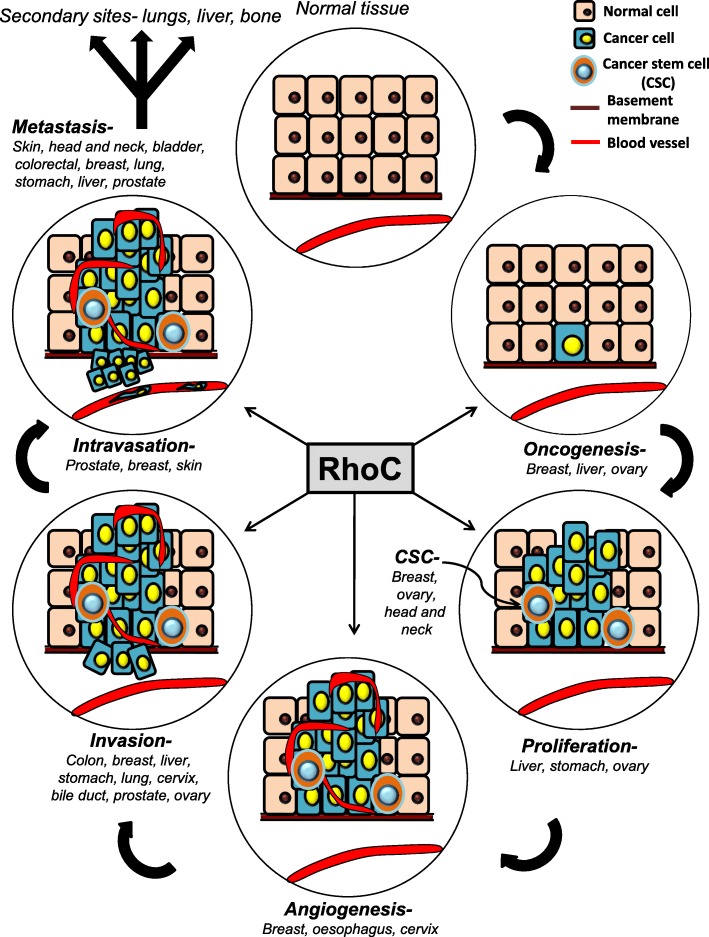
An illustration depicting the diverse roles of RhoC in various aspects of cancer progression: RhoC significantly contributes to cancer initiation, proliferation, stemness maintenance, angiogenesis, invasion, intravasation, and metastasis across numerous tumor models, as shown

Not only does RhoC regulate tumor growth, EMT, migration, invasion, and metastasis, it also regulates angiogenesis in tumors. Vasculogenesis and angiogenesis are controlled by angiogenic factors, such as VEGF-A [78]. In the physiological context, RhoC stimulates the proliferation of human umbilical vein endothelial cells (HUVECs) by stabilizing beta-catenin, which in turn enhances cyclin D1 expression. Cyclin-D1 subsequently drives cell-cycle progression [79]. Apart from proliferation, RhoC also regulates various angiogenic features like pseudopod formation and migration ability in HUVECs and MVECs (myeloma vascular endothelial cells) via ROCK and MAPK signaling [80]. RhoC has been shown to regulate angiogenesis in breast cancer [73, 81], where it modulates the expression of VEGF, fibroblast growth factor-basic (bFGF), interleukin-6 and interleukin-8, which are important in angiogenesis [81]. Similarly, RhoC may promote VEGF expression in oesophageal squamous cell carcinoma, thus regulating angiogenesis [82]. In cervical cancer, conditioned media from SiHa cells stably over-expressing the RhoC gene resulted in increased in vitro tube formation by HUVEC cells. Additionally, immunohistochemical analysis of clinical specimens revealed that RhoC and VEGF were expressed in the same areas of tumor sections [56]. Cancers promote the development of an alternate vascular system (known as vasculogenic mimicry) to support its growth and proliferation. RhoC and its effector ROCK2 have been shown to play important roles in this context by activation of the ERK and MMP pathways in the hepatocellular carcinoma model [83]. The importance of RhoC to carcinoma progression was exemplified by Hakem et al. Using a mouse model, they showed that RhoC was dispensable for post-natal development, however it’s depletion reduced metastasis [21]. The disseminated tumor cells survived in blood vessels until they relocated to a distant site and formed metastases. The ability to survive in these conditions is an important attribute of metastatic tumor cells. We have earlier published that RhoC is also involved in anoikis resistance [56], which may aid the cells to survive for prolonged durations in suspension in blood. As summarized in Fig. 2, RhoC therefore plays an important role at various stages of tumor progression.

Intriguingly, while RhoC has been highly implicated in several aspects of carcinoma progression, there is no report of a mutation associated with this gene [84]. Analysis of the COSMIC database suggests that mutations of RhoC in cancer are very rare. Only 60 unique samples out of 47,923 showed mutations. In total, there were only 17 missense mutations, 7 silent mutations and 1 deletion mutation included in the database. Interestingly, these mutations are scattered across the protein domains, indicating that they are unlikely to be driver mutations and are most likely passenger mutations.

### RhoC in Cancer stem cells

The phenotypic and functional heterogeneity observed among cells within the same tumor represents one of the greatest challenges in cancer therapeutics and has caused confounding clinical outcomes, as it results in heterogeneous therapy response. The plasticity of tumor cells enables them to adapt and survive at different stages of tumor progression in a dynamically changing microenvironment, beginning from the site of tumor initiation and ending at a distant metastatic site. Such plastic tumor cells exhibit several stem-like characteristics, such as self-renewal, high drug efflux capacity and better DNA repair, and are thus termed cancer stem cells (CSCs) [85, 86].

**Fig. 3:**
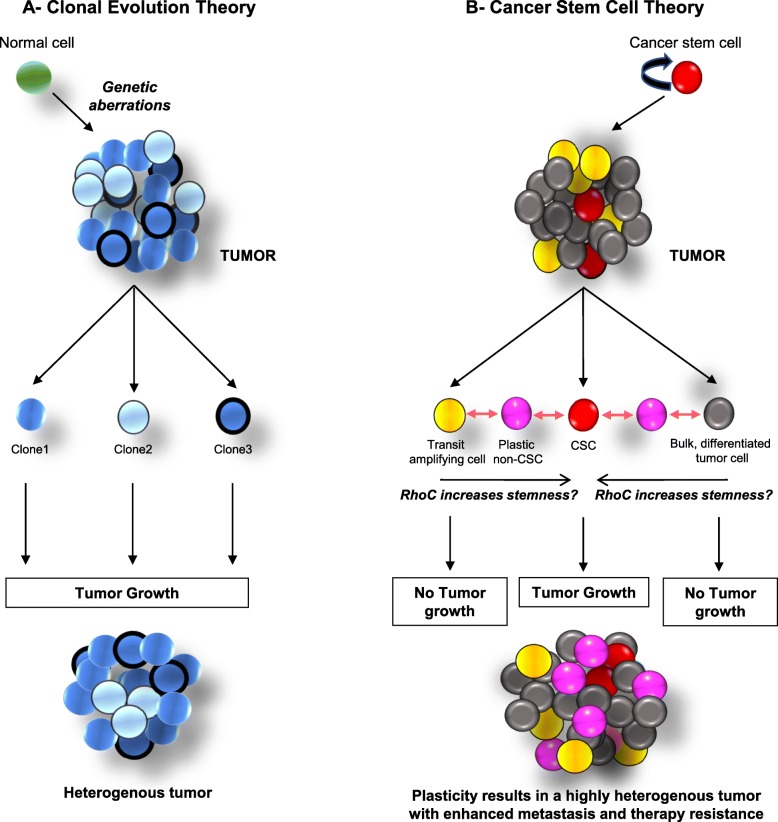
Tumor induction models and the possible role of RhoC: The clonal evolution theory postulates that genetic abnormalities lead to tumor formation, with every clone of cells thus produced being equally capable of regenerating the tumor (**a**). On the contrary, the cancer stem cell theory proposes the presence of a minute sub-population known as cancer stem cells (CSCs), which alone hold the potential for resurgence of the various populations that constitute the tumor. This includes the differentiated tumor bulk, transit amplifying cells which are mildly pluripotent and proliferative and an intermediate substantially pluripotent “plastic” state (**b**). These cellular states are highly dynamic with cells being capable of constantly moving from one state to another. We propose that RhoC, with it’s involvement in multiple tumor phenotypes could play a pivotal role in regulating this “switch” via it’s downstream effectors

Tumor formation is broadly believed to adhere to the stochastic/clonal evolution model or the hierarchical/classical CSC model [87]. The clonal evolution model attributes cancer initiation to genetic abnormalities within a normal cell. According to the clonal evolution theory, these aberrations lead to a heterogenous tumor pool consisting of multiple clones, each of them being equally proficient at giving rise to a tumor. The classical CSC model on the other hand, entrusts tumor induction capability solely to the CSC population. This theory believes that a cancer stem cell gives rise to transit amplifying cells, which further give rise to the differentiated tumor bulk. Recent findings have led scientists to believe that this model is not unidirectional, but is in fact highly dynamic and plastic, allowing for interconversion of these states via differentiation and dedifferentiation resulting in a complex, heterogenous tumor [87]. An outline of these models has been illustrated in Fig. 3. Several reports cumulatively suggest that RhoC regulates numerous steps of tumor progression, including proliferation [56, 88, 89], EMT [62, 63], invasion [61, 90, 91], intravasation [56, 92], extravasation [92], anoikis resistance [56], angiogenesis [56, 92] and metastasis [58, 61, 93]. Resultantly, it is apt to believe that RhoC may be involved in regulating or maintaining tumor plasticity, which endows adaptability at every stage of tumor progression. Plasticity is known to be an inherent feature of stem cells and in line with this, recent research has shown that RhoC is involved in maintenance of the stemness phenotype.

An important finding by Rosenthal et al. indicates a strong correlation between RhoC and ALDH, a breast cancer stem cell (BCSC) marker [93]. Using the aggressive BCSC cell line SUM149, Rosenthal et al. show that cells with active ALDH (ALDH+) have higher levels of RhoC than those with inactive ALDH (ALDH−). Tumorigenicity studies utilizing a limiting number of 50 cells in mice resulted in no induction of tumors in mice injected with ALDH+/shRhoC cells, whereas 5 out of 9 mice with ALDH+/scrambled cells formed tumors. Moreover, incidences of lung metastases were found to be around five times higher in mice injected with ALDH+/scrambled cells compared to those injected with ALDH+/shRhoC cells, indicating the stem-like property of cells containing RhoC. Finally, a tissue microarray of breast cancer samples from 136 patients indicated a high correlation between RhoC and ALDH1, further supporting RhoC’s association with ALDH.

The role of RhoC in CSC maintenance has also been illustrated in head and neck squamous cell carcinoma (HNSCC) by Islam et al. [94]. Using UM-SCC-1 and UM-SCC-47 cell lines, they show that the siRNA mediated inhibition of RhoC led to decreased expression of ALDH, CD44, Oct3/4, Sox2, and Nanog, in addition to a diminished formation of tumorspheres. Further, Islam et al. determine that tumorspheres have increased levels of RhoC and genes associated with stemness compared to cells grown as monolayers, whereas the inhibition of RhoC leads to a reduction in the expression of stemness genes, which points towards RhoC’s possible role in CSC induction. Islam et al. then demonstrate that RhoC leads to stemness induction in head and neck cancer by activation of STAT3 via IL-6. In a study by Sang et al., ovarian cancer stem cells (OCSCs) were sorted using the CD117 marker from A2780-PM and A2780-PTX-PM, two drug-resistant and invasive ovarian cancer cell lines [90]. These OCSCs were found to have elevated expressions of RhoC. The MTT (3-(4,5-dimethylthiazol-2-yl)-2,5-diphenyltetrazolium bromide) assay revealed that cells inhibited for RhoC had decreased cell proliferation and drug resistance. Further, inhibition of RhoC by RhoC-specific siRNA led to decreased expression of stemness markers like CD133 and CD117, as observed by real-time quantitative PCR, suggesting a possible role of RhoC in the formation of OCSCs. It may be thus appropriate to deduce that overexpression of RhoC results in enhanced plasticity/stemness of cancer cells (Fig. 3).

### RhoC and resistance to Cancer therapy

Therapy resistance occurs when the tumor stops responding to treatments, such as radiation, chemotherapy and other forms of targeted therapy. Current approaches primarily kill treatment-sensitive cells, while the therapy-resistant cells survive and expand over time to cause recurrence. In order to ensure a complete cure, it is important to eliminate all cancer cells, including the therapy-resistant ones, which have now come to be known as CSCs. Indeed, recent reports suggest a vast number of molecular mechanisms that regulate and contribute to resistance. RhoC has also been shown to contribute to therapy resistance in some tumor models. Interestingly, a seminal article by Mani et al., which reported that EMT induces stemness in cancer cells, opened up a new understanding of CSCs and their targeting [95]. This study showed that transformed human mammary epithelial cells that had undergone EMT formed tumors much more efficiently and had stemness properties. Therefore, this study opens up avenues to explore opportunities to target pathways that regulate EMT and resultantly eliminate CSCs, the culprit for recurrence and metastasis.

RhoC is one such protein whose role in EMT induction and regulation is well documented. It has been demonstrated that RhoC along with LIM Domain Kinase 2 (LIMK2) is a direct target of p53 during chemotherapy [96]. Kawata et al. demonstrated that RhoC may have a role to play in endocrine therapy resistance, a significant barrier to successful prostate cancer treatment [97]. Two years later, the same group reported that RhoC was up-regulated in breast cancer samples post chemotherapy treatment, indicating increased RhoC levels in the chemoresistant population. They also showed a correlation between RhoC expression and reduced E-cadherin levels, pointing towards a possible role for RhoC in EMT, thereby leading to therapy resistance in breast cancer [98]. Research from our laboratory (unpublished data) shows that RhoC and its downstream effector, ROCK2 regulates the radioresistance in cervical cancer.

Several groups have reported that RhoC inhibitors have a profound effect on carcinoma phenotypes in vitro, using both cell lines and tumor biopsy-derived cells. Wenandy et al. attempted to understand RhoC’s clinical application and found that RhoC has a Human Leukocyte Antigen-A3 (HLA-A3) restricted epitope, which is recognized by cytotoxic T cells. Wenandy et al. propose that RhoC may serve as a target for anti-cancer immunotherapy [99]. Inhibitors of 3-hydroxy-3-methyl-glutaryl-coenzyme A reductase (HMG-CoA reductase), commonly known as statins, have been widely used to understand the function of this molecule. The HMG-CoA pathway produces geranylgeranyl pyrophosphate (GGPP) and Farnesyl pyrophosphate (FPP) as intermediate products, which are important for activation of RhoGTPases [100]. Interestingly, the use of farnesyltransferase inhibitors (FTI) has been shown to have a profound effect on tumor phenotype. Treatment of IBC cells with FTI showed a reversal of RhoC-induced phenotypes like anoikis resistance, motility, and invasion [73]. Collisson EA et al. proposed statins as a primary prophylaxis for melanoma, demonstrating a reduction in RhoC activation upon treatment with atorvastatin, consequently leading to inhibition of invasion and metastasis [100]. Another study reported that a combinatorial use of atorvastatin and celecoxib, in vitro, resulted in an induction of cell cycle arrest and apoptosis in colon cancer cells [101]. Atorvastatin-mediated inhibition of RhoC also blocked metastasis in head and neck cancer cells, in vitro [102]. Encouraging reports indicate that use of the statin group of drugs reduces incidence of esophageal cancer [103]. A study by Kaushal et al. showed that antiRhoC siRNA led to decreased invasion, motility, and migration of the breast cancer cell lines SUM149 and MDA-MB-231, suggesting that RhoC is a potential therapeutic target [104]. This group went on to further design “smart” nanoparticles that delivered anti-RhoC siRNA into breast cancer cells, thereby successfully impeding migration and invasion [105].

Despite a series of convincing reports on RhoC’s role in various tumor phenotypes, it has not been developed further as a prognostic marker or therapeutic target. There have been attempts to use inhibitors, such as atorvastatin, to understand its function, but further development has not been reported. Considering its extensive contribution to carcinomas and their progression, it is important to initiate studies to define RhoC as a potential therapeutic target.

## Conclusion

CSCs have the ability to evade therapy, repair and survive under stressful conditions, such as hypoxia. These cells also have EMT properties, coupled with the ability to invade and migrate. Resistance to therapy has also been attributed to CSCs in several tumors. Given that CSCs are an important subset of the tumor and can elicit various tumor phenotypes, it is important to develop targets against these cells for better cancer care. The ideal target for such adaptive and plastic cells would be a molecular pathway that is important for CSC maintenance and regulates several functional attributes of these cells. The available literature suggests that RhoC has a major contribution in CSC maintenance. The role of RhoC in carcinoma progression has been well studied and reported. This molecule has a central role in most of the reported tumor phenotypes, with recent reports pointing to its possible role in the stemness of cancer cells. Given the evidence implicating RhoC in various aspects of tumor progression, this molecule seems to be an ideal druggable target. However, the three Rho GTPases: RhoA, RhoB, and RhoC show an 85% amino acid sequence identity, leaving little room for the development of a specific inhibitor to RhoC alone [15]. Nevertheless, considering that the role of RhoC in tumor progression is overwhelming, efforts must be steered towards developing siRNA, antibodies, or small molecule-based inhibitors of RhoC. It is, therefore, of prime importance to thoroughly explore the application of this molecule in cancer prognostication, in order to efficiently tackle the disease.

## Data Availability

Data sharing not applicable to this article as no datasets were generated or analysed during the current study.

## Abbreviations

BCSC: Breast cancer stem cell
bFGF: Fibroblast growth factor-basic
Cas/Rac1: Crk-associated substrate/Ras-Related C3 Botulinum Toxin Substrate 1
CSCs: Cancer stem cells
EGFR: Epidermal Growth Factor Receptor
EMT: Epithelial to mesenchymal transition
FAK: Focal Adhesion Kinase
FMNL3: Formin-like 3
FPP: Farnesyl pyrophosphate
GAPs: GTPase Activating Proteins
GDIs: Guanine Dissociation Inhibitors
GDP: Guanosine Diphosphate
GEFs: Guanine Nucleotide Exchange Factors
GGPP: Geranylgeranyl pyrophosphate
GTPases: Guanosine Triphosphatases
HLA-A3: Human Leukocyte Antigen-A3
HNSCC: Head and neck squamous cell carcinoma
HUVECs: Human umbilical vein endothelial cells
IBC: Inflammatory breast cancer
LIMK2: LIM Domain Kinase 2
MAPK: Mitogen Activated Protein Kinase
mDia: Diaphanous Related Formin
miRNAs: MicroRNAs
MMP9: Matrix Metalloprotease 9
MRTF: Myocardin-related transcription factors
MVECs: Myeloma vascular endothelial cells
NFκB: Nuclear Factor kappa-light-chain-enhancer of activated B cells
OCSCs: Ovarian cancer stem cells
PI3K/AKT: Phosphoinositide 3 kinase**/**AKT Serine Threonine Kinase
PYK2: Protein-Tyrosine Kinase 2
RhoC: Ras homolog gene family member C
ROCK: Rho Associated Coiled-Coil Containing Protein Kinase
TGF-beta: Tumor Growth Factor-beta
VEGF: Vascular Endothelial Growth Factor
